# Associations Between Employment Changes and Mental Health: US Data From During the COVID-19 Pandemic

**DOI:** 10.3389/fpsyg.2021.631510

**Published:** 2021-02-11

**Authors:** Cillian P. McDowell, Matthew P. Herring, Jeni Lansing, Cassandra S. Brower, Jacob D. Meyer

**Affiliations:** ^1^The Irish Longitudinal Study of Ageing, Trinity College Dublin, The University of Dublin, Dublin, Ireland; ^2^School of Medicine, Trinity College Dublin, The University of Dublin, Dublin, Ireland; ^3^Physical Activity for Health Research Cluster, Health Research Institute, University of Limerick, Limerick, Ireland; ^4^Department of Physical Education and Sport Sciences, University of Limerick, Limerick, Ireland; ^5^Department of Kinesiology, Iowa State University, Ames, IA, United States

**Keywords:** coronavirus, employment, working from home (WFH), depression, anxiety, lonelineness, stress, positive mental health

## Abstract

**Objectives:** To examine associations of changing employment conditions, specifically switching to working from home (WFH) or job loss, with mental health, using data collected during the COVID-19 pandemic.

**Methods:** Data from 2,301 US adults in employment prior to COVID-19 were collected April 3rd−7th, 2020. Participants reported whether their employment remained unchanged, they were WFH when they had not been before, or they had lost their job due to the pandemic. Outcomes were symptoms of depression, anxiety, stress, loneliness, and positive mental health (PMH) assessed using validated questionnaires. Linear regression quantified associations of employment changes with mental health outcomes, controlling for age, sex, race, BMI, smoking status, screen time, physical activity, marital status, chronic conditions, and current COVID-19 containment strategies being followed.

**Results:** Compared to participants whose employment remained unchanged, those who switched to WFH did not differ in any measures of mental health (all *p* ≥ 0.200). Participants who had lost their job reported higher symptoms of depression (*g* = −0.200, 95%CI = −0.333 to −0.067; *p* = 0.003), anxiety (*g* = −0.212, −0.363 to −0.061; *p* = 0.008), and stress (*g* = −0.348, −0.482 to −0.214; *p* < 0.001), and lower PMH (*g* = −0.212, −0.347 to −0.078; *p* = 0.002). Loneliness did not differ between groups (*p* = 0.087).

**Conclusion:** This study demonstrates (1) that concerns around potential adverse mental health effects, particularly increases in loneliness, should not preclude WFH in the general population, while considering each individual's personal circumstances, and (2) the acute adverse association of job loss with mental health. Tailored and sensitive interventions may be required to prevent deteriorations in mental health associated with job loss during periods of societal stress.

## Introduction

The novel coronavirus (COVID-19) has rapidly altered life globally, transforming how, and even whether, people work. In the US, all 50 states declared a state of emergency by March 16th, 2020 and introduced diverse measures designed to limit the disease transmission to prevent critically overburdening healthcare systems (Gostin and Wiley, 2020). Many businesses closed temporarily or permanently and many people switched to working from home (WFH). Data from the National Bureau of Economic Research suggest that between February and May 2020 over one third of the labor force switched to remote work, resulting in about half of American workers WFH, and 10.1% had been laid off (Brynjolfsson et al., 2020). The pandemic-driven changes may foreshadow more lasting effects on the organization of work; however, the potential mental health impacts of these rapid changes in employment is not well-understood.

It is plausible that shifting to WFH may be associated with improved mental health, with increased free-time and potential scope for improved work-life balance. Alternatively, trying to live and work in the same environment may be a source of stress, and isolation from co-workers a cause for loneliness. Moreover, the deleterious mental health effects of job losses are well-known (Tiggemann and Winefield, 1984), and people whose employment remains unchanged are likely experiencing their own mental health challenges. Indeed, the mental health effects of the COVID-19 pandemic are likely to be long lasting, with evidence already demonstrating the substantial psychological burden during the outbreak (Brooks et al., 2020; Pfefferbaum and North, 2020). Therefore, this study aimed to explore switching to WFH and loss of employment were associated with impaired mental health early April in the US.

## Methods

### Study Characteristics

This study uses cross-sectional data from The COVID-19 and Well-being (Cov-Well) Study. This sample has been utilized in previous studies (McDowell et al., 2020; Meyer et al., 2020; Cindrich et al., 2021) and details of the methodology employed by Cov-Well are fully described elsewhere (Meyer et al., 2020). Briefly, Cov-Well is a survey including cross-sectional and longitudinal components which were approved as an exempt project by Iowa State University's Institutional Review Board (approval #: 20-144-00). Convenience sampling using mass emails that included a link to an anonymous online survey to Iowa State University students, faculty, staff, and alumni, snowball sampling, and posts to social media pages were used to recruit potential participants. Participants considered for the current study completed the survey April 3rd–April 7th, 2020 and were employed prior to COVID-19 (*n* = 2,454). Participants with missing employment, mental health, and covariate data were excluded (*n* = 64; 2.7%), as were those with implausible body mass index (BMI; i.e., 4 standard deviations above the mean) and activity values (i.e., >16 h/day of physical activity or >20 h/day of physical activity and sitting; *n* = 89; 3.6%), leaving a final sample of 2,301.

### COVID-19-Related Employment Changes

Participants were asked “what is the impact of the recent events on your work life?” with possible answers “no change in work,” “working from home, when I was not before,” and “lost employment in relation to pandemic.”

### Mental Health

Depressive symptoms were assessed by the 21-item Beck Depression Inventory-II, excluding the suicidality question (range: 0–63) (Beck et al., 1996). Anxiety symptoms were assessed by the 21-item Beck Anxiety Inventory (range: 0–63) (Beck et al., 1988). Stress was assessed by the 4-item Perceived Stress Scale (range: 0–16) (Lee, 2012). Loneliness was assessed by the 3-item Loneliness scale (range: 3–9) (Hughes et al., 2004). Positive mental health (PMH) was assessed by the Short Warwick-Edinburgh Mental Well-being Scale (range: 7–35) (Haver et al., 2015).

### Covariates

Covariates were age (10-year categories), sex (male, female, or transgender), race (white or other), BMI, smoking status (current smoker or not), screen time (more or <8-h/day), physical activity (meeting recommended guidelines or not), marital status (married/in a relationship, widowed, separated/divorced, or never married), chronic conditions (summed into three categories: 0, 1, and ≥2), and current public health restrictions (quarantined/required to quarantine/self-isolating, under a shelter-in-place/stay-at-home order, or social distancing). Education was assessed but removed from primary analyses due to multicollinearity.

### Analyses

Data were analyzed in Stata version 14.2. Multivariable linear regression quantified adjusted associations of COVID-19-related employment changes with continuous symptoms of depression, anxiety, stress, loneliness, and PMH. Multicollinearity was determined as likely if two covariates had a correlation ≥0.8, the mean variance inflation factor (VIF) was ≥6, or the highest individual VIF was ≥10. Consequently, education was excluded from analyses. Robust standard errors, which are robust to heteroscedasticity, were used in the multivariable linear regression. Statistical significance was set at *p* < 0.01 to adjust for multiple testing. Hedges' *g* effect sizes and associated 95% confidence intervals (95% CIs) were subsequently calculated such that worse mental health was represented as a negative effect size. E-values were subsequently calculated to assess how robust associations were to potential uncontrolled confounding (Borenstein et al., 2009; Ding and VanderWeele, 2016; Vander Weele, 2017). The E-value denotes the minimum strength of association that an uncontrolled confounder would need to have with both the predictor and outcome to fully explain away their associations, conditional on the measured covariates (Vander Weele and Ding, 2017; Haneuse et al., 2019).

## Results

**Figure 1:**
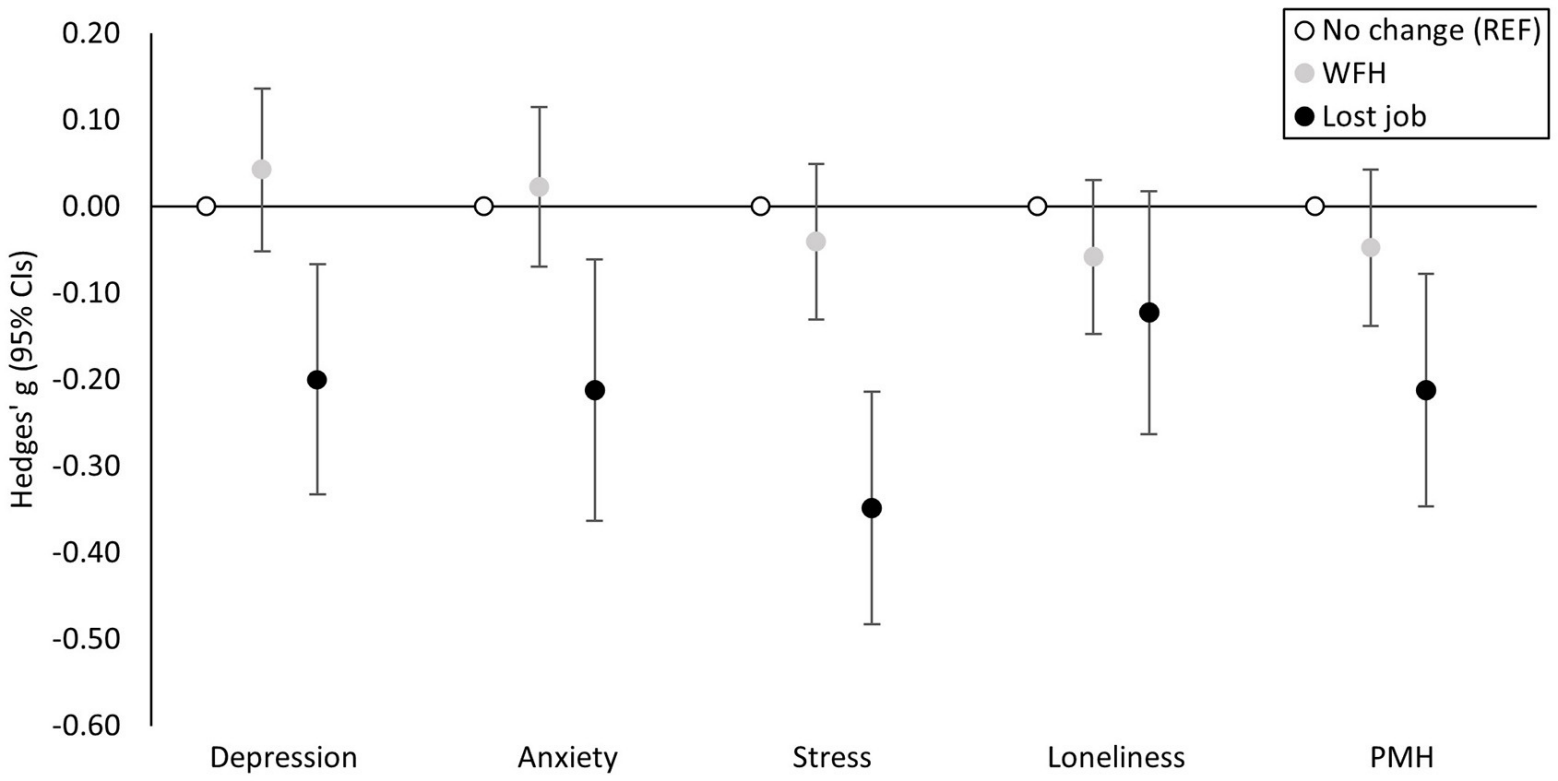
Hedges' *g* effect sizes and associated 95% confidence intervals (CIs) demonstrating the magnitude of the adjusted association between working from home (WFH) and job loss (compared to unchanged employment) and mental health such that poorer mental health is illustrated by a negative effect size. PMH, positive mental health; REF, reference category.

Participants (*n* = 2,301; 66% female) were evenly dispersed across age categories from 18 to 74, with 78 participants aged ≥75 years, and were predominantly white (92%), educated (84.6% college graduate or above), and overweight (BMI = 26.95 ± 5.90), but mostly (74%) without chronic conditions. Most (54%) were WFH when they were not before, 34% reported no change in employment, and 12% reported loss of employment. Mean ± SD outcome scores were: depression (10.29 ± 8.56), anxiety (7.96 ± 8.38), stress (6.36 ± 2.95), loneliness (5.19 ± 1.80), and PMH (23.73 ± 4.52). Results from primary analyses are presented in Figure 1. Compared to participants whose employment remained unchanged, those who switched to WFH did not differ in any measures of mental health (all *p* ≥ 0.200). Participants who had lost their job reported higher symptoms of depression (*g* = −0.200, 95%CI = −0.333 to −0.067; *p* = 0.003), anxiety (*g* = −0.212, −0.363 to −0.061; *p* = 0.008), and stress (*g* = −0.348, −0.482 to −0.214; *p* < 0.001), and lower PMH (*g* = −0.212, −0.347 to −0.078; *p* = 0.002). E-values for these associations and their confidence interval closest to the null are as follows: depression [E = 1.69 (1.32)], anxiety [E = 1.72 (1.30)], stress [E = 2.09 (1.73)], and PMH [E = 1.72 (1.35)]. This means that an uncontrolled confounder that was associated with both job loss and depressive symptoms by a magnitude equivalent to a risk ratio of 1.69 could nullify the observed association between job loss and depressive symptoms, or 1.32 for its lower confidence interval, but weaker confounding could not. Similarly, an uncontrolled confounder that was associated with both job loss and anxiety symptoms by a magnitude equivalent to a risk ratio of 1.72 at a minimum could explain away the observed association between job loss and anxiety symptoms. Hedges' *g* effect sizes approximately equivalent to these risk ratio values for the primary associations vary between *g* = 0.58 to *g* = 0.81, and *g* = 0.29 to *g* = 0.60 for lower confidence intervals. Loneliness did not differ between groups (*p* = 0.087).

## Discussion

This study quantified associations of changing employment conditions, specifically switching to working from home or loss of job, with mental health, using data collected during the COVID-19 pandemic. Compared to participants whose employment remained unchanged, those who switched to WFH did not report different mental health. Those who lost their jobs reported higher symptoms of depression, anxiety, and stress, and lower PMH. Data were collected April 3rd-April 7th, 2020, by which time substantial employment-related changes had manifested (Brynjolfsson et al., 2020). These findings may have important implications for limiting the mental health impact of the COVID-19 pandemic, especially with new evidence demonstrating bidirectional associations between mental illness and COVID-19 infection which may augment already existing health inequalities (Taquet et al., 2020; Yao et al., 2020; Wang et al., 2021). However, they also have important implications beyond the pandemic as work practices may shift and more people switch to WFH.

Consistent with National Bureau of Economic Research data from a similar time-period (Brynjolfsson et al., 2020), most participants reported WFH when they were not before and 12% reported losing employment due to the pandemic. The finding that loss of employment was associated with adverse mental health is also consistent with previous findings (Tiggemann and Winefield, 1984). Moreover, according to the E-values these findings appear unlikely to be nullified by an uncontrolled confounding variable. An uncontrolled confounder would require associations between *g* = 0.58 and *g* = 0.81 (depending on the outcome) with both job loss and a given mental health outcome in order to nullify the observed association. Although plausible, this seems unlikely as most major potential sources of confounding were controlled for in analyses.

Although not assessed in the current study, it seems likely that the associations between job loss and mental health are explained by financial concerns and concerns regarding sustained unemployment and subsequent impact on their lives (e.g., loss of house, no access to healthcare, etc). Ensuring adequate access to mental health care among people in the US, in particular those who have lost their jobs, is likely essential to avoid prolonged mental health impacts from the pandemic. Additionally, it is plausible that these associations are mediated by self-esteem as job loss is associated with reduced self-esteem which may precede increases in depressive and anxiety symptoms (Tiggemann and Winefield, 1984; Sowislo and Orth, 2013).

Evidence has demonstrated the effects of the COVID-19 pandemic on lifestyle behaviors (e.g., increases in sedentary behavior and reductions in physical activity), and it is plausible that employment-related changes due to COVID-19 may contribute to these changes (McDowell et al., 2020; Meyer et al., 2020). Increases in sedentary behaviors, in particular screen time, may be associated with negative mental health outcomes, while engaging in physical activity may be protective of mental health (McDowell et al., 2019; Huang et al., 2020; Dishman et al., 2021). Although the potential mediating role of lifestyle behaviors was not examined in the current study, maintaining a healthy lifestyle may play an important role in sustaining mental health in the midst of job fluctuations (Venkatesh and Edirappuli, 2020; World Health Organization, 2020).

This study has several limitations. The cross-sectional design precludes inference of causality. The convenience sample is predominantly well-educated and white and so not entirely reflective of the total US population. Although well-validated questionnaires were used, behaviors, and mental health outcomes were self-reported and so potentially subject to misreporting. Finally, socioeconomic status was not adjusted for in analyses as education, an indicator of socioeconomic status that is associated with health and mortality (Rosengren, 2019), was excluded from the models due to multicollinearity. Although results did not materially differ when it was included, socioeconomic status is a multidimensional construct that can be indicated by additional factors such as occupational group, wealth, and place of residence.

### Implications

This study demonstrates that concerns around potential adverse mental health effects, particularly increases in loneliness, should not preclude WFH in the general population, while considering each individual's personal circumstances. Secondly, given the large increase in unemployment resulting from COVID-19, along with the anticipated economic downturn, ensuring that systems are in place to address the potential increased need for mental health resources is a matter of urgency.

## Data Availability Statement

The raw data supporting the conclusions of this article will be made available by the authors, without undue reservation. Requests to access these data should be directed to Jacob Meyer, jdmeyer3@iastate.edu.

## Ethics Statement

The studies involving human participants were reviewed and approved by Iowa State University's Institutional Review Board. The patients/participants provided their written informed consent to participate in this study.

## Author Contributions

CMcD, JM, and MH: analysis and interpretation of data and drafting of the manuscript. All authors are study concept and design and revision of the manuscript.

## Conflict of Interest

The authors declare that the research was conducted in the absence of any commercial or financial relationships that could be construed as a potential conflict of interest.
